# Efficacy and safety of camrelizumab plus apatinib for solid tumors: a meta-analysis

**DOI:** 10.3389/fimmu.2025.1653429

**Published:** 2025-09-02

**Authors:** Ping Yu, Xiaoyu Song, Yue Li, Xuanlong Chen, Yuan Zhou, Fei Jiang

**Affiliations:** 1 Department of Pharmacy, Hubei Provincial Clinical Research Center for Umbilical Cord Blood Hematopoietic Stem Cells, Taihe Hospital, Hubei University of Medicine, Shiyan, Hubei, China; 2 Departmentof Ultrasound Medicine, Taihe Hospital, Hubei University of Medicine, Shiyan, Hubei, China; 3 Department of Respiratory, Taihe Hospital of Shiyan, Hubei University of Medicine, Shiyan, Hubei, China

**Keywords:** camrelizumab, apatinib, solid tumors, meta-analysis, efficacy

## Abstract

**Objective:**

This study used meta-analysis to explore the efficacy and safety of camrelizumab plus apatinib in the treatment of solid tumors.

**Methods:**

PubMed, Embase, web of science, and Cochrane library databases were searched for this study, and the searches were conducted from database creation to August 13, 2025. The minors (methodological index for nonrandomized studies) score was used to evaluate the quality of the included studies, and the meta-package in R4.3.3 was used to analyze the data.

**Result:**

The analysis of 35 publications involving 2224 patients revealed various adverse events and survival outcomes. Adverse events of any grade included anemia (ES=0.446), diarrhea (ES=0.217), hypertension (ES=0.478), proteinuria (ES=0.402), and fatigue (ES=0.328). For grade1-2, adverse events, the effect sizes ranged from 0.146to 0.306. For grade ≥3 adverse events, the effect sizes ranged from 0.004 to 0.065. The Objective Response Rate (ORR) was 40.0%, with a Disease Control Rate (DCR) of 78.0%. Overall Survival (OS) rates at 6, 12, and 24 months were 79.0%, 46.5%, and 16.0%, respectively. Progression-Free Survival (PFS) rates at the same intervals were 48.4%, 19.8%, and 6.7%.

**Conclusion:**

According to the results of this meta-analysis, although the camrelizumab plus apatinib treatment regimen demonstrated certain efficacy in the short term, due to the significant limitations of this study, more high-quality, multicenter, large-sample randomized controlled studies are needed in the future to corroborate our conclusions.

## 1 Introduction

Various solid neoplasms encompass a range of malignant tumors like those affecting the bladder, prostate, breast, colon, and kidneys (1, 2). Annually, approximately 10 million individuals succumb to malignant neoplasms globally, positioning them as a leading cause of mortality (3). Their high incidence rates coupled with treatment complexities pose significant threats to human life and well-being, impacting economic prosperity and societal equilibrium (4, 5). All nations are actively exploring innovative approaches to combat these neoplasms. Presently, standard tumor therapies encompass surgical interventions, chemotherapy, radiotherapy, targeted therapy, and traditional Chinese medicine (6). In recent years, advancements in therapeutic modalities have prolonged the survival durations of patients with tumors. Nonetheless, challenges such as drug resistance emergence, treatment-induced recurrence susceptibility, and other clinical hurdles persist (7). Immunotherapy, heralded for its capacity to activate and augment the immune system against tumor cells, has garnered substantial attention (8). Its merits include enduring therapeutic efficacy, proficient prevention of tumor relapse, and prospective curative outcomes. Nevertheless, immunotherapy confronts obstacles like immune evasion and suboptimal efficacy in managing solid neoplasms (9, 10).

Recent years have witnessed the emergence of anti-angiogenic targeted therapy and immunotherapy as focal points of research, offering novel treatment avenues for malignant tumors (11). Camrelizumab, a humanized anti-PD1 IgG4 monoclonal antibody, functions as an immune checkpoint inhibitor by binding with high affinity to PD-1 (B7-H1), thus inhibiting its interaction and enhancing the proliferation and cytokine secretion of tumor antigen-specific T cells (12). Clinical findings indicate a significant enhancement in the overall survival of lung cancer patients with the integration of chemotherapy and camrelizumab (13). The profound exploration of tumor biology has underscored the pivotal role of angiogenesis in tumor progression, invasion, and metastasis. Tumor angiogenesis, governed by vascular endothelial growth factor (VEGF) overexpression, serves as a critical determinant in tumor proliferation, differentiation, and metastasis (14, 15). Apatinib is a selective vascular endothelial growth factor receptor-2 (VEGFR-2) tyrosine kinase inhibitor. VEGFR-2 is a key receptor for tumor neovascularization. By inhibiting VEGFR-2, Apatinib can effectively inhibit tumor angiogenesis and improve the tumor microenvironment, thereby reducing the supply of nutrients and oxygen to tumor cells and limiting tumor growth and metastasis. Apatinib can not only inhibit the growth of tumor blood vessels, but also affect the tumor microenvironment, reduce immunosuppression, increase the infiltration of immune cells, and enhance the effect of tumor immunotherapy (16, 17). In the treatment of a variety of solid tumors, Apatinib has shown significant clinical efficacy, especially in patients with chemotherapy-resistant gastric cancer, non-small cell lung cancer and other tumors, which can significantly improve the survival and quality of life of patients. The application of Apatinib has been approved in many countries, including China, and has become one of the standard treatment options for advanced gastric cancer and other malignant tumors. Despite the significant anti-tumor effect of Apatinib, its efficacy as monotherapy is still limited in some patients resulting in unstable efficacy. Therefore, the combination of apatinib with immune checkpoint inhibitors has become a research hotspot with a view to exerting greater synergistic effects. Nonetheless, solitary administration of apatinib often prompts tumor resistance, a challenge ameliorated by the enduring tumor-suppressive effects of camrelizumab. Furthermore, the constrained efficacy of camrelizumab can be enhanced through apatinib-mediated modulation of the body’s immune status and the tumor’s immune response (18). Preclinical and clinical studies suggest that carilizumab combined with apatinib may have synergistic effects. On the one hand, by inhibiting tumor angiogenesis, apatinib may improve the tumor microenvironment and enhance T-cell infiltration, thus enhancing the anti-tumor effect of PD-1 monoclonal antibody; on the other hand, the activating effect of immunotherapy may also enhance the anti-angiogenic effect of apatinib, making its inhibitory effect on tumors more durable (19). The combined treatment modality of immune checkpoint inhibitors and anti-angiogenic agents has shown potential in a wide range of tumor types. The combination of carilizumab and apatinib could theoretically enhance the efficacy through several mechanisms: on the one hand, carilizumab activates the body’s immune system by releasing the inhibitory state of T-cells, while apatinib enhances the infiltration of immune cells by inhibiting tumor angiogenesis and improving the tumor microenvironment. On the other hand, the tumor angiogenesis inhibited by apatinib can make the tumor cells more exposed to the surveillance of the immune system, thus improving the effect of immunotherapy. In addition, the immune-activating effects of carilizumab may help to overcome resistance to abatinib when used alone, making combination therapy more effective. Several clinical trials have investigated the efficacy of the combination of carelizumab and apatinib in a variety of solid tumors. For example, in patients with gastric cancer and non-small cell lung cancer, the combination of carelizumab and apatinib significantly improved the objective remission rate (ORR) and disease control rate (DCR), while prolonging progression-free survival (PFS) and overall survival (OS). However, the combination regimen also poses certain challenges, mainly in the form of increased toxicities, especially hypertension, proteinuria, and immune-related adverse events.

The aim of this study is to quantitatively analyze the efficacy and safety of the combination of carilizumab and apatinib in solid tumors by integrating the available clinical data and using systematic evaluation and meta-analysis methods. We hope that this analysis will provide more comprehensive and accurate evidence to inform clinicians and help them make more precise decisions in treatment selection. Further studies may also provide a new theoretical basis for the combined application of tumor immunotherapy and targeted therapy, promote the development of individualized treatment strategies, and ultimately improve the therapeutic efficacy and quality of life of patients with solid tumors.

## 2 Materials and methods

### 2.1 Literature search

PubMed, Embase, Web of Science, and Cochrane Library databases were systematically queried from their inception to August 13, 2025, for this investigation. The search strategy incorporated the terms “neoplasm,” “cancer,” “apatinib,” and “camrelizumab.” Detailed search history is delineated in **Supplementary Table S1**.

### 2.2 Inclusion and exclusion criteria

The study’s eligibility criteria encompassed solid tumors such as bladder, prostate, breast, colon, and renal site tumors. The interventions comprised administering camrelizumab intravenously and apatinib orally. Primary outcome focused on adverse events, while secondary outcomes included Objective Response Rate (ORR), Disease Control Rate (DCR), Overall Survival (OS), and Progression-Free Survival (PFS). Encompassed study types ranged from randomized controlled trials to retrospective and single-arm studies. The study’s exclusion criteria comprised duplicate publications, reviews, conference abstracts, case reports, lack of full-text availability, and absence of data.

### 2.3 Data extraction

Data extraction for this study utilized Excel sheets, and the literature underwent thorough screening by two authors adhering to rigorous inclusion and exclusion criteria. Any disparities were diligently resolved through consultation or by seeking a third-party opinion to achieve consensus. Extracted data encompassed study details, publication year, sample size, gender distribution, age demographics, tumor characteristics, intervention modalities, and outcomes.

### 2.4 Quality assessment of included studies

For randomized controlled trials, the assessment of bias risk utilized the Cochrane’s Randomized Clinical Trials Risk of Bias Tool 2.0 (RoB2) (20). This tool was independently applied by two investigators. In cases of disagreement between investigators, consensus was achieved through consultation with a third investigator. The evaluation encompassed various aspects including the randomization process, adherence to expected interventions, handling of missing outcome data, selection of outcome measures, and reporting of outcomes. Subsequently, studies were categorized into low, moderate, or high risk of bias. In contrast, single-arm and retrospective studies underwent quality assessment using the Newcastle-Ottawa Scale (NOS) (21). This tool assigns scores ranging from 0 to 9, with scores of 0–3 indicating poor quality, 4–6 indicating fair quality, and 7–9 indicating good quality studies. Any discrepancies in scoring were resolved through consensus.

### 2.5 Grade of evidence

To determine the quality of our results, we selected the Graded Recommendations Assessment Development and Evaluation (GRADE) system to evaluate the evidence (22) for methodological quality. We considered five factors that could reduce the quality of the evidence, including study limitations, inconsistent findings, inconclusive direct evidence, inaccurate or wide confidence intervals, and publication bias. In addition, three factors that could reduce the quality of evidence were reviewed, namely effect size, possible confounding factors, and dose-effect relationships. A comprehensive description of the quality of evidence for each parameter data is provided. grade results are available in **Supplementary Table S2**.

### 2.6 Statistical analysis

Heterogeneity was assessed utilizing the Cochran Q test alongside the I2 statistic. Selection of effect models was based on the I^2^ statistic: when I^2^ exceeded 50%, a random effects model was employed; otherwise, a fixed effects model was utilized. Subgroup analysis was conducted to investigate potential sources of heterogeneity. Publication bias was examined using a funnel plot. These analyses were conducted using R, version 4.3.3. Statistical significance was defined as p < 0.05.

## 3 Result

### 3.1 Literature screening results

**Figure 1:**
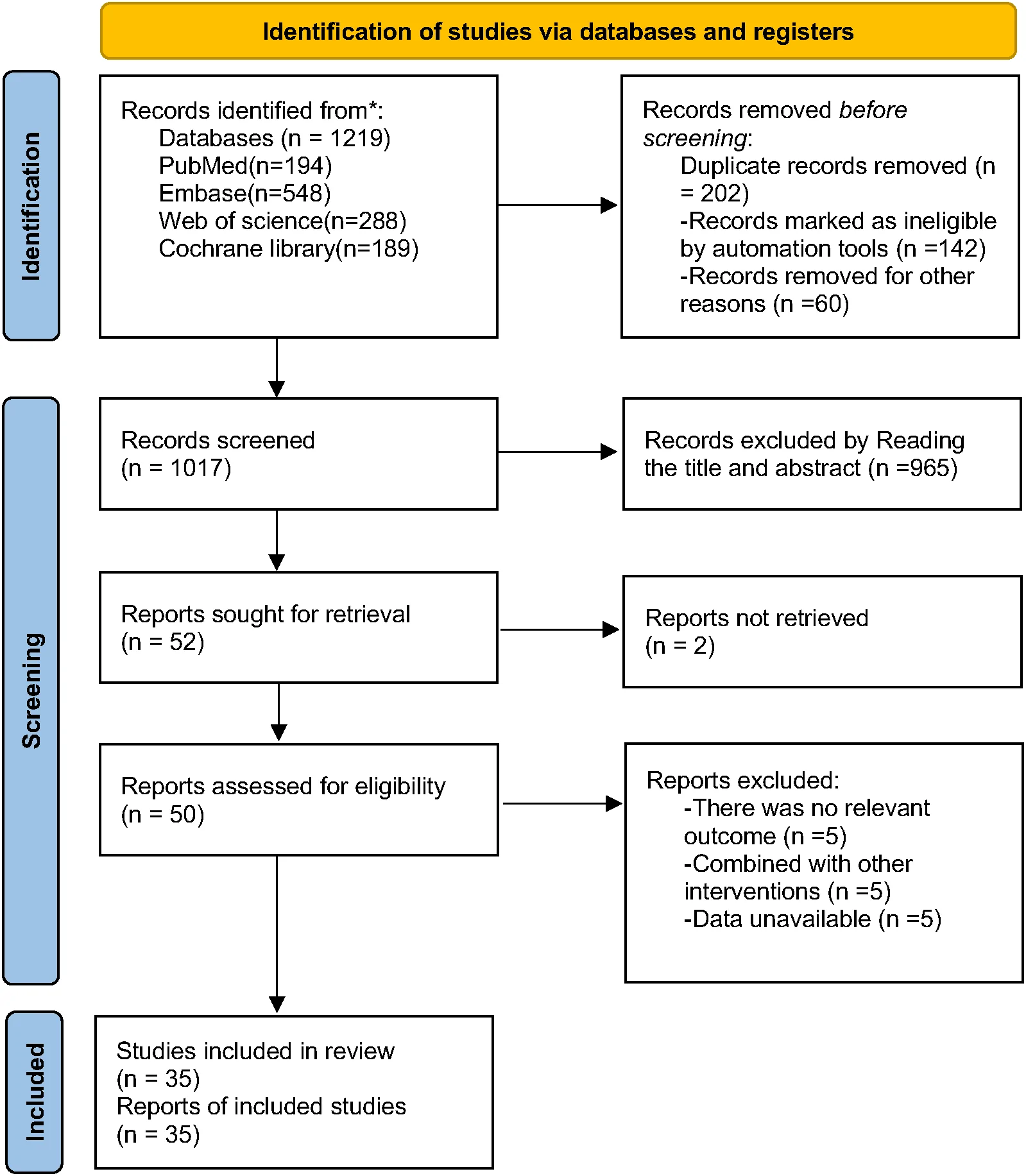
Literature search flow chart.

An initial search of the literature identified 1219 articles (PubMed (n=194), Embase (n=548), Web of science (n=288), Cochrane library (n=189)), which were removed by removing duplicates (n=202), removed by reading titles and abstracts (n=965), and removed by reading the full text (n=15), resulting in the inclusion of 35 studies (23–57), the specific literature search flowchart is shown in **Figure 1**. 35 publications (2242 patients) included 2 randomized controlled studies (33, 39), 2 retrospective studies (23, 31), 31single arm studies (24–30, 32, 34–38, 40–57), and the category types included breast, lung, gastric, nasopharyngeal, hepatocellular, colorectal, and oral cancers. camrelizumab dose 200mg intravenously and apatinib 250mg orally. The specific literature characterization table is shown in **Table 1**.

**Table 1:** Basic characteristics of the literature.

Study	Year	Sample size	Gender(M/F)	Mean age (years)	Tumor type	Previous treatment	Intervention	Outcome
Lin (33)	2024	CA+CT:51 CT:53	77/27	CA+CT:63 CT:63	advanced gastric cancer	NO	C: IV; camrelizumab; 200mg A: apatinib; orally; 250mg	ORR; DCR; AEs
Qin (39)	2023	CA+CT:272 CT:271	457/86	CA+CT:58 CT:56	hepatocellular carcinoma	NO	C: IV; camrelizumab; 200mg A: apatinib; orally; 250mg	OS; PFS; ORR; DCR; AEs
Chen (24)	2024	34	32/2	58.5	advanced gastric cancer	NO	C: IV; camrelizumab; 200mg A: apatinib; orally; 250mg	OS; PFS; ORR; DCR; AEs
Cheng (25)	2021	20	0/20	33.5	gestational trophoblastic neoplasia	YES	C: IV; camrelizumab; 200mg A: apatinib; orally; 250mg	ORR; AEs
Ding (26)	2023	58	46/12	48	nasopharyngeal carcinoma	YES	C: IV; camrelizumab; 200mg A: apatinib; orally; 250mg	OS; PFS; ORR; DCR; AEs
Gao (28)	2022	43	22/21	55	non-small cell lung cancer	NO	C: IV; camrelizumab; 200mg A: apatinib; orally; 250mg	OS; PFS; ORR; DCR; AEs
Ju (29)	2022	21	12/9	56.4	oral squamous cell carcinoma	NO	C: IV; camrelizumab; 200mg A: apatinib; orally; 250mg	ORR; DCR; AEs
Liang (32)	2024	49	37/12	46	nasopharyngeal carcinoma	NO	C: IV; camrelizumab; 200mg A: apatinib; orally; 250mg	OS; ORR; DCR; AEs
Ma (35)	2022	19	12/7	63	metastatic gastric cancer	YES	C: IV; camrelizumab; 200mg A: apatinib; orally; 250mg	OS; PFS; ORR; DCR; AEs
Mei (36)	2021	28	21/7	52	hepatocellular carcinoma	YES	C: IV; camrelizumab; 200mg A: apatinib; orally; 250mg	OS; PFS; ORR; DCR; AEs
Meng (37)	2022	52	42/10	63	esophageal squamous cell carcinoma	YES	C: IV; camrelizumab; 200mg A: apatinib; orally; 250mg	OS; PFS; ORR; DCR; AEs
Mo (38)	2023	26	22/4	49	nasopharyngeal carcinoma	YES	C: IV; camrelizumab; 200mg A: apatinib; orally; 250mg	OS; PFS; ORR; DCR; AEs
Qu (40)	2024	29	18/11	65	esophageal squamous cell carcinoma	NO	C: IV; camrelizumab; 200mg A: apatinib; orally; 250mg	OS; PFS; ORR; DCR; AEs
Ren (41)	2024	41	35/6	64	non-small cell lung cancer	NO	C: IV; camrelizumab; 200mg A: apatinib; orally; 250mg	OS; PFS; ORR; DCR; AEs
Wang (43)	2021	21	11/10	60	Biliary tract cancers	YES	C: IV; camrelizumab; 200mg A: apatinib; orally; 250mg	OS; PFS; ORR; DCR; AEs
Wang (44)	2023	30	16/14	65	acral melanoma	NO	C: IV; camrelizumab; 200mg A: apatinib; orally; 250mg	OS; PFS; ORR; DCR; AEs
Xia (46)	2022	18	17/1	54.7	hepatocellular carcinoma	NO	C: IV; camrelizumab; 200mg A: apatinib; orally; 250mg	ORR; DCR; AEs
Xu (48)	2021	190	169/21	52	hepatocellular carcinoma	NO	C: IV; camrelizumab; 200mg A: apatinib; orally; 250mg	OS; PFS; ORR; DCR; AEs
Yao (49)	2023	29	13/16	56	lung adenocarcinoma	YES	C: IV; camrelizumab; 200mg A: apatinib; orally; 250mg	OS; PFS; ORR; DCR; AEs
Yuan (51)	2020	63	58/5	48.7	hepatocellular carcinoma	YES	C: IV; camrelizumab; 200mg A: apatinib; orally; 250mg	OS; PFS; ORR; DCR; AEs
Yuan (52)	2023	72	56/16	45	nasopharyngeal carcinoma	NO	C: IV; camrelizumab; 200mg A: apatinib; orally; 250mg	ORR; DCR; AEs
Zeng (53)	2021	45	35/10	52	hepatocellular carcinoma	NO	C: IV; camrelizumab; 200mg A: apatinib; orally; 250mg	OS; PFS; ORR; DCR; AEs
Zhang (54)	2020	30	23/7	55	esophageal squamous cell carcinoma	YES	C: IV; camrelizumab; 200mg A: apatinib; orally; 250mg	ORR; DCR; AEs
Zhou (56)	2021	105	79/26	58	non-small cell lung cancer	YES	C: IV; camrelizumab; 200mg A: apatinib; orally; 250mg	OS; PFS; ORR; DCR; AEs
Fan (27)	2021	59	20/39	51	Extensive-Stage small cell lung cancer	NO	C: IV; camrelizumab; 200mg A: apatinib; orally; 250mg	OS; PFS; ORR; DCR; AEs
Lan (30)	2020	45	0/45	51	Cervical Cancer	NO	C: IV; camrelizumab; 200mg A: apatinib; orally; 250mg	OS; PFS; ORR; DCR; AEs
Liu (34)	2020	40	0/40	45.5	breast cancer	YES	C: IV; camrelizumab; 200mg A: apatinib; orally; 250mg	OS; PFS; ORR; DCR; AEs
Xie (47)	2020	41	30/11	19	osteosarcoma	NO	C: IV; camrelizumab; 200mg A: apatinib; orally; 250mg	OS; PFS; AEs
Chen (23)	2023	CA+CT:71 CT:72	125/18	57	hepatocellular carcinoma	NO	C: IV; camrelizumab; 200mg A: apatinib; orally; 250mg	OS; PFS; ORR; DCR; AEs
Li (31)	2022	CA+CT:31 CT:33	38/26	59.5	colorectal cancer	YES	C: IV; camrelizumab; 200mg A: apatinib; orally; 250mg	OS; PFS; ORR; DCR; AEs
Tian (42)	2024	36	0/36	60	endometrial cancer	YES	C: IV; camrelizumab; 200mg A: apatinib; orally; 250mg	OS; PFS; ORR; DCR; AEs
Xia (45)	2024	21	17/4	67	non-small cell lung cancer	NO	C: IV; camrelizumab; 200mg A: apatinib; orally; 250mg	OS; ORR; DCR; AEs
Yu (50)	2024	52	29/23	52.5	colorectal cancer	NO	C: IV; camrelizumab; 200mg A: apatinib; orally; 250mg	ORR; DCR; AEs
Zhao (55)	2024	32	18/14	62	Melanoma	NO	C: IV; camrelizumab; 200mg A: apatinib; orally; 250mg	OS; PFS; ORR; DCR; AEs
Zhu (57)	2024	21	8/13	48	adrenocortical carcinoma	YES	C: IV; camrelizumab; 200mg A: apatinib; orally; 250mg	OS; PFS; ORR; DCR; AEs

M/F, male/female; C, camrelizumab; A, apatinib; ORR, Objective response rate; DCR, Disease control rate; AEs, adverse events; OS, Overall survival; PFS, Progression-free survival.

### 3.2 Risk of bias in inclusion literature

For the 2 randomized controlled studies, both clearly accounted for the randomized multiple consumption method, and blinding. It was therefore evaluated as low risk, and the specific risk of bias results are shown in **Supplementary Figure S1**.For the single-arm and retrospective studies the NOS score was used, and the studies scored between 6 and 8, as shown in **Supplementary Table S3**.

### 3.3 Results of meta-analysis

#### 3.3.1 Adverse events

The adverse events assessed in this study encompassed anemia, diarrhea, hypertension, proteinuria, and fatigue. The analysis results (**Table 2**) indicated the following effect sizes and corresponding 95% confidence intervals (CI) for any grade adverse events, the analysis revealed the following effect sizes and corresponding 95% confidence intervals (CI): anemia (ES=0.446, 95% CI: 0.301, 0.545), diarrhea (ES=0.217, 95% CI: 0.156, 0.368), hypertension (ES=0.478, 95% CI: 0.356, 0.674), proteinuria (ES=0.402, 95% CI: 0.284, 0.458), and fatigue (ES=0.328, 95% CI: 0.256, 0.409). Regarding grade 1–2 adverse events, the effect sizes and 95% confidence intervals were as follows: anemia (ES=0.146, 95% CI: 0.109, 0.345), diarrhea (ES=0.177, 95% CI: 0.136, 0.217), hypertension (ES=0.351, 95% CI: 0.296, 0.439), proteinuria (ES=0.360, 95% CI: 0.267, 0.458), and fatigue (ES=0.291, 95% CI: 0.211, 0.377). For adverse events of grade ≥3, the effect sizes and 95% confidence intervals were: anemia (ES=0.017, 95% CI: 0.011, 0.027), diarrhea (ES=0.004, 95% CI: 0.002, 0.024), hypertension (ES=0.065, 95% CI: 0.043, 0.179), proteinuria (ES=0.031, 95% CI: 0.008, 0.073), and fatigue (ES=0.019, 95% CI: 0.007, 0.047).

**Table 2:** Results of meta-analysis of adverse events.

Adverse event	Any grade					Grade 1-2					Grade≥3				
	Study	Heterogeneity		ES (95%CI)	P	Study	Heterogeneity		ES (95%CI)	P	Study	Heterogeneity		ES (95%CI)	P
		P	I^2^ (%)				P	I^2^ (%)				P	I^2^ (%)		
Anemia	25	0.001	96.2	0.446 (0.301,0.545)	0.001	25	0.001	90	0.146 (0.109,0.3455)	0.001	25	0.01	64	0.017 (0.011, 0.027)	0.01
Diarrhea	24	0.001	81.2	0.217 (0.156,0.368)	0.003	24	0.001	84	0.177 (0.136,0.217)	0.003	24	0.465	0	0.004 (0.002, 0.024)	0.001
Hypertension	32	0.001	91.2	0.478 (0.356, 0.674)	0.001	32	0.001	86	0.351 (0.296, 0.439)	0.001	32	0.001	92	0.065 (0.043, 0.179)	0.001
Proteinuria	33	0.001	93.2	0.402 (0.284, 0.458)	0.001	33	0.001	93	0.360 (0.267, 0.458)	0.001	33	0.001	87	0.031 (0.008, 0.073)	0.002
Fatigue	28	0.001	84.3	0.328 (0.256, 0.409)	0.001	28	0.001	76	0.291 (0.211, 0.377)	0.001	28	0.058	41	0.019 (0.007, 0.047)	0.001

#### 3.3.2 Objective response rate

**Figure 2:**
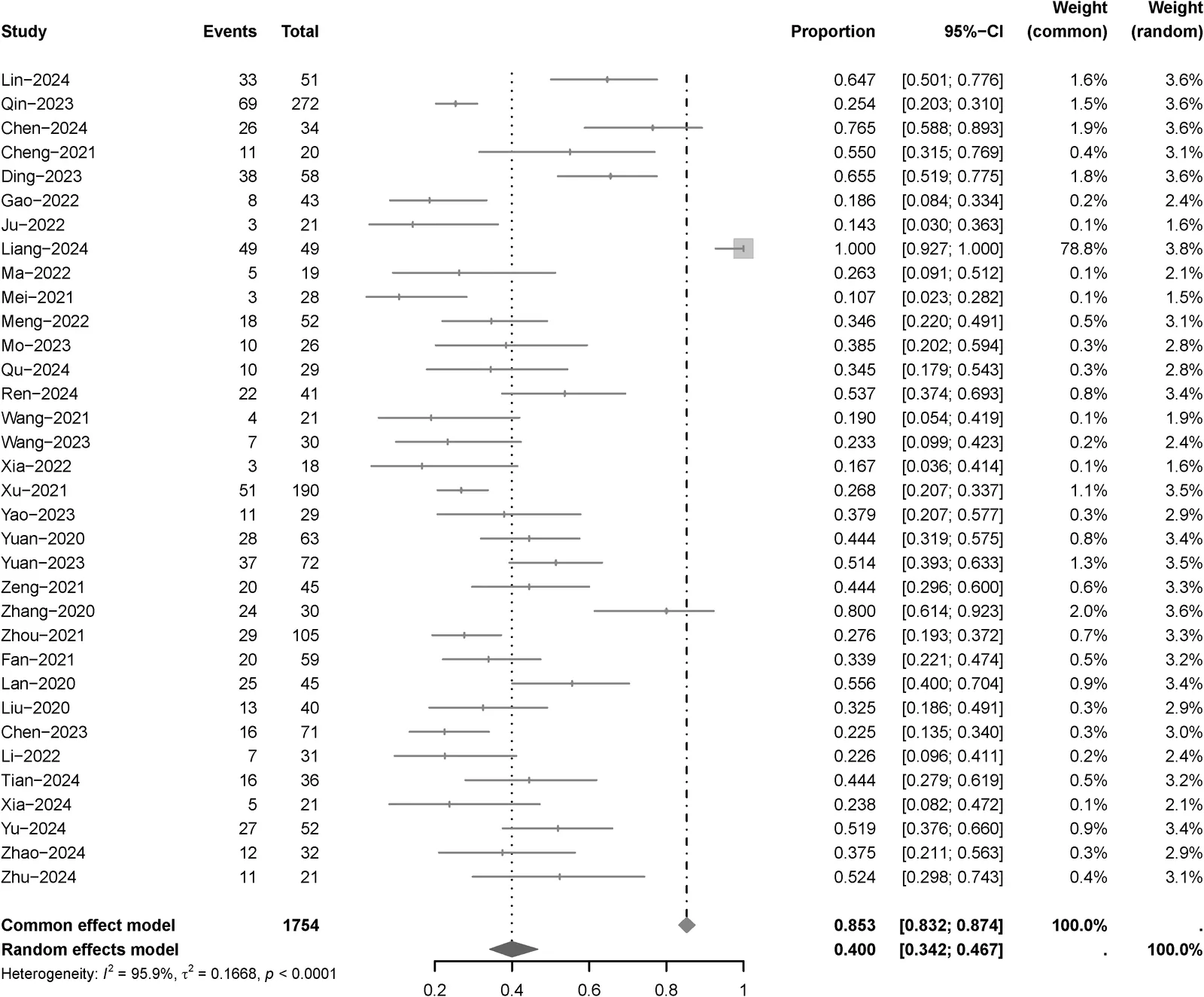
Forest plot of objective response rate meta-analysis.

Thirty-five articles examined ORR, with a heterogeneity test revealing an I^2^ value of 95.9%. Utilizing a random-effects model for analysis, the results (**Figure 2**) indicated an ORR of 40.0% (95% CI: 34.2%-46.7%), following the administration of camrelizumab plus apatinib. Due to significant heterogeneity in this indicator, a sensitivity analysis was conducted through iterative exclusion of individual articles. The results (**Supplementary Figure S2**) suggested minimal sensitivity, indicating relative stability in the analytical outcomes. Subgroup analysis (**Supplementary Figure S3**) based on tumor type revealed the following ORR: 56.7% (95% CI: 33.8%-95.0%) for advanced gastric cancer, 28.6% (95% CI: 21.5%-38.1%) for hepatocellular carcinoma, and 63.0% (95% CI: 43.1%-92.1%) for nasopharyngeal carcinoma. Subgroup analysis (**Supplementary Figure S4**) based on different lines of treatment revealed the following ORR: 39.6% (95% CI: 31.6%-49.6%) for frontline; 39.6% (95% CI: 31.8%-49.3%) for no frontline.

#### 3.3.3 Disease control rate

**Figure 3:**
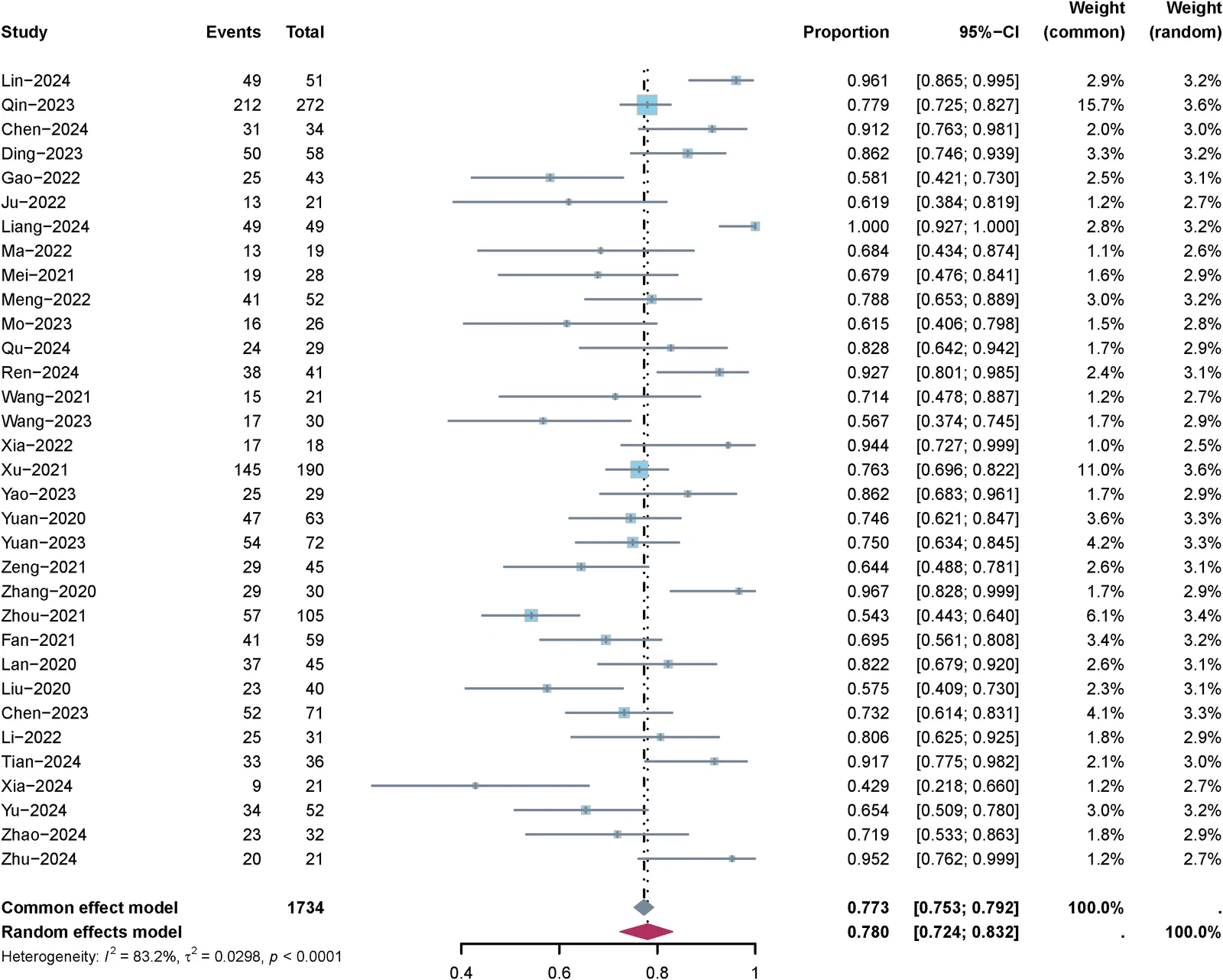
Forest plot of disease control rate meta-analysis.

Thirty-five examined DCR, with a heterogeneity test revealing an I^2^ value of 83.2%. Utilizing a random-effects model for analysis, the results (**Figure 3**) indicated an DCR of 78.0% (95% CI: 72.4%-83.2%), following the administration of camrelizumab plus apatinib. Due to significant heterogeneity in this indicator, a sensitivity analysis was conducted through iterative exclusion of individual articles. The results (**Supplementary Figure S5**) suggested minimal sensitivity, indicating relative stability in the analytical outcomes. Subgroup analysis (**Supplementary Figure S6**) based on tumor type revealed the following DCR: 88.1% (95% CI: 70.5%-98.4%) for advanced gastric cancer, 76.0% (95% CI: 72.8%-79.2%) for hepatocellular carcinoma, and 85.5% (95%CI: 61.5%-98.8%) for nasopharyngeal carcinoma. Subgroup analysis (**Supplementary Figure S7**) based on different lines of treatment revealed the following DCR: 78.0% (95% CI: 70.0%-85.2%) for frontline; 77.9% (95% CI: 69.7%-85.1%) for no frontline.

#### 3.3.4 Overall survival

**Figure 4:**
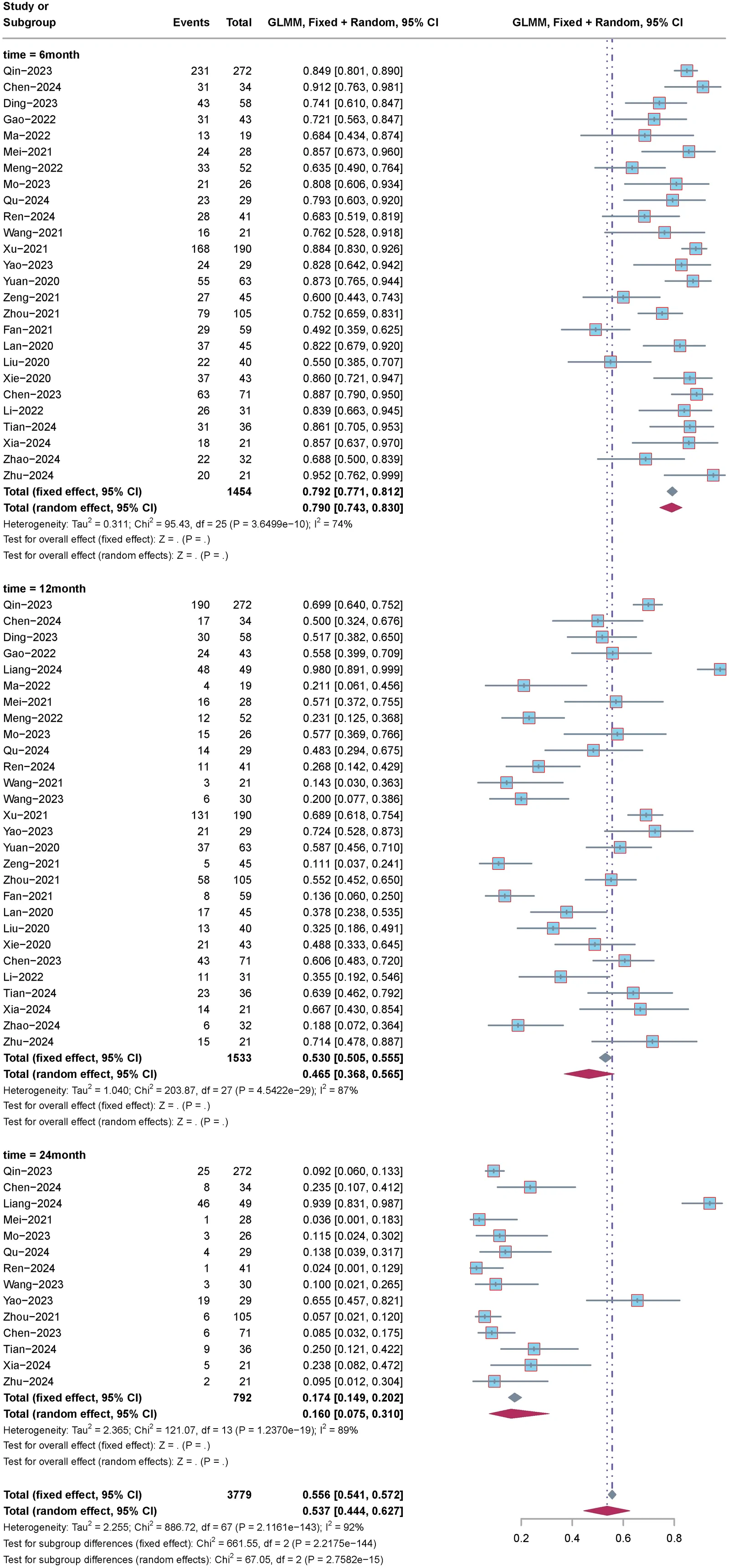
Forest plot of overall survival meta-analysis.

Subgroup analysis of OS based on time intervals, including 6 months, 12 months, and 24 months, was conducted. The analysis results (**Figure 4**) indicated the following: For 6-month OS: Effect size (ES) was 79%, with a 95% confidence interval (CI) of 74.3% to 83.0%, and heterogeneity (I^2^) was 74%. For 12-month OS: ES was 46.5%, with a 95%CI of 36.8% to 56.5%, and heterogeneity (I^2^) was 87%. For 24-month OS: ES was 16.9%, with a 95%CI of 7.5% to 31.0%, and heterogeneity (I^2^) was 89%.

#### 3.3.5 Progression free survival

**Figure 5:**
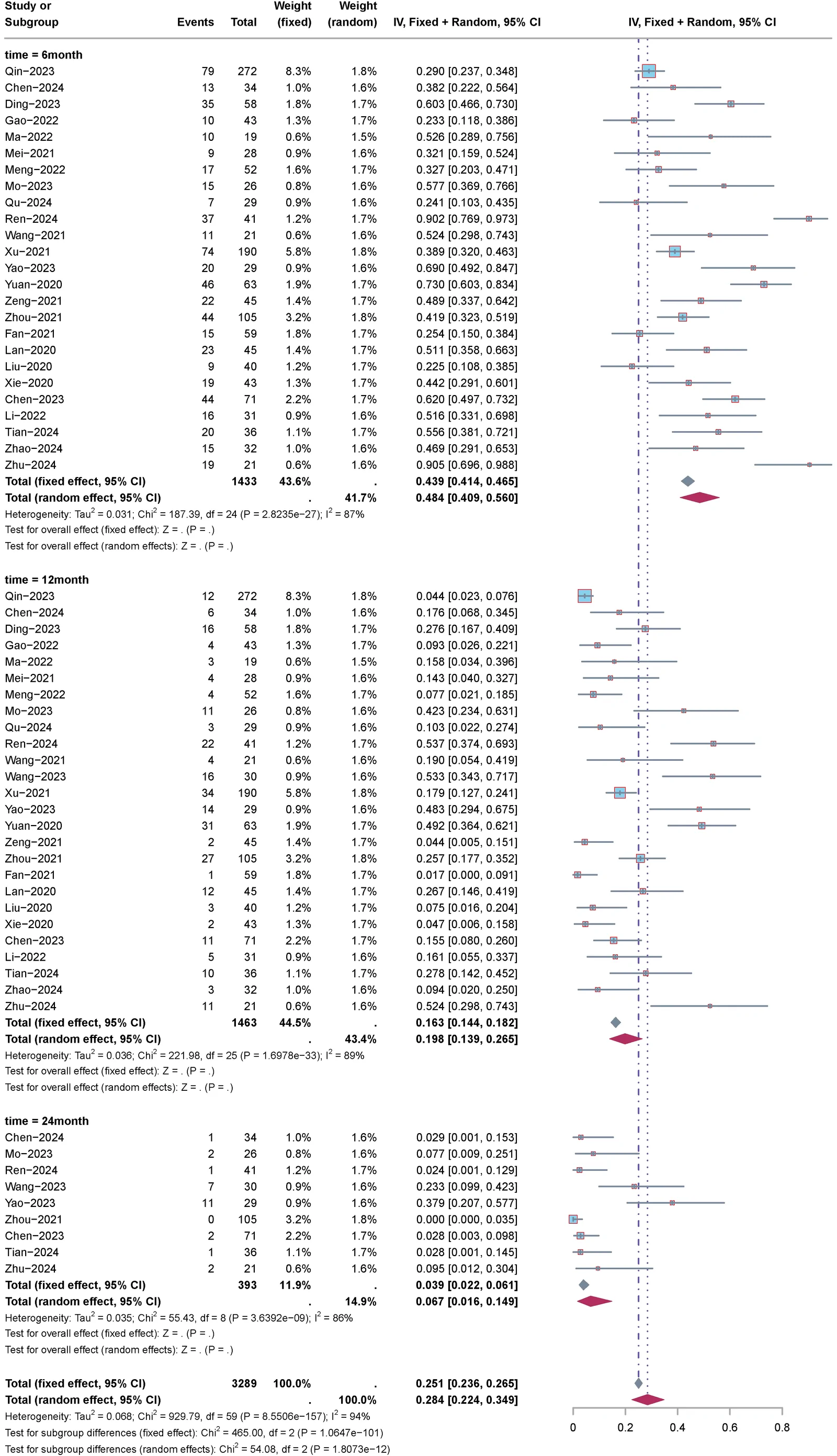
Forest plot of progression free survival meta-analysis.

Subgroup analysis of PFS based on time intervals, including 6 months, 12 months, and 24 months, was conducted. The analysis results (**Figure 5**) indicated the following: For 6-month PFS: ES was 48.4%, with a 95% confidence interval (CI) of 40.9% to 56.0%, and heterogeneity (I^2^) was 87%. For 12-month PFS: ES was 19.8%, with a 95%CI of 13.9% to 26.5%, and heterogeneity (I^2^) was 89%. For 24-month PFS: ES was 6.7%, with a 95%CI of 1.6% to 14.9%, and heterogeneity (I^2^) was 86%.

### 3.4 Publication bias

We assessed publication bias using funnel plots for OS, PFS, ORR, DCR, and the results (**Supplementary Figures S8–S11**) suggested that publication bias was more likely for the above outcomes.

### 3.5 Results of meta regression analysis

Due to the high heterogeneity of this study, meta-regression was used to explore the sources of heterogeneity. The results (**Supplementary Table S4**) suggest that the sources of heterogeneity for OS, PFS, ORR, and DCR may be tumor type.

## 4 Discussion

To our knowledge, this study represents the inaugural utilization of a meta-analysis approach to investigate the efficacy and safety of camrelizumab plus apatinib in solid tumor treatment. The meta-analysis revealed adverse events at any grade, including anemia (ES=0.446), diarrhea (ES=0.217), hypertension (ES=0.478), proteinuria (ES=0.402), and fatigue (ES=0.328). Regarding grade 1–2 adverse events, the effect sizes were: anemia (ES=0.146), diarrhea (ES=0.177), hypertension (ES=0.351), proteinuria (ES=0.360), and fatigue (ES=0.291). For grade ≥3 adverse events, the effect sizes were: anemia (ES=0.017), diarrhea (ES=0.004), hypertension (ES=0.065), proteinuria (ES=0.031), and fatigue (ES=0.019). Furthermore, the study reported an objective response rate (ORR) of 40.0% (95% CI: 34.2%-46.7%) and a disease control rate (DCR) of 78.0% (95% CI: 72.4%-83.2%). Regarding overall survival (OS), rates at 6, 12, and 24 months were determined to be 79% (95% CI: 74.3%-83.0%), 46.5% (95% CI: 36.8%-56.5%), and 16.9% (95% CI: 7.5%-31.0%), respectively. Progression-free survival (PFS) for the corresponding time periods was found to be 48.4% (95% CI: 40.9%-56.0%), 19.8% (95% CI: 13.9%-26.5%), and 6.7% (95% CI: 1.6%-14.9%), respectively.

Existing research underscores the pivotal role of the tumor microenvironment in facilitating tumor growth and progression. Within this milieu, the tumor immune microenvironment and tumor angiogenesis emerge as critical components. Antiangiogenic agents have been shown to augment PD-1/PD-L1 therapy by stimulating tumor endothelial micro vessels, thereby enhancing T lymphocyte infiltration and activity (58, 59). This synergistic interaction broadens the therapeutic scope for diseases that are refractory to standalone PD-1/PD-L1 inhibitors. Conversely, PD-1/PD-L1 blockade enhances the sensitivity and extends the efficacy of antiangiogenic agents, exemplifying a synergistic effect (60). Notably, hepatocellular carcinoma, nasopharyngeal carcinoma, and gastric carcinoma, exemplifying inflammation-associated and vascular-rich tumors, present promising targets for combined PD-1 inhibitor and antiangiogenic agent therapy. In the context of this study, the combination of camrelizumab and apatinib exhibited favorable outcomes in hepatocellular carcinoma, gastric carcinoma, and nasopharyngeal carcinoma. These results signify a notable improvement compared to phase 2 clinical trials of camrelizumab monotherapy in terms of ORR (29.0% vs. 14.7%) and DCR (72.0% vs. 44.2%) (61). Although ORR and DCR are two different indicators, they are closely related and provide complementary information about the efficacy of treatment. In many cases, the goal of tumor therapy is not only to achieve a complete remission, but to be able to control disease progression and improve the patient’s quality of life. Therefore, although the ORR results may appear to be low, the high DCR values indicate that the treatment is effective in slowing down the progression of the disease in most patients, which is clinically important. In the present study, the DCR was significantly higher than the ORR, suggesting that most patients were able to maintain stable rather than fully progressive disease after receiving the combination of camrelizumab and apatinib. This phenomenon was particularly evident in patients with advanced gastric and nasopharyngeal cancers, where the DCR was higher, reflecting the potential of this therapeutic combination in controlling disease. Analysis of the ORR and DCR provides a more comprehensive understanding of the potential of this treatment combination in different tumor types. Immunotherapy typically works by activating the immune system to suppress tumor growth rather than directly causing significant tumor shrinkage (62). Therefore, despite the relatively low ORR observed in this study, the high DCR indicates that the treatment effectively stabilized the disease. Biological responses to immunotherapy often exhibit time delays, as the immune system gradually exerts its effects. Consequently, while no obvious tumor shrinkage may be observed in the short term, long-term control of disease progression is achievable. Additionally, tumor heterogeneity and immune escape mechanisms may prevent complete tumor elimination, but overall disease control is maintained through immune surveillance. This mechanism is a common manifestation in immunotherapy, particularly in certain tumor microenvironments or cases involving complex apoptosis mechanisms (63). Although both ORR and DCR showed more positive efficacy results overall, high heterogeneity (ORR I²=96%, DCR I²=90%) remains a distinctive feature of this study. This heterogeneity mainly stems from differences in study design, patient selection, treatment regimen and tumor type. The high heterogeneity may make the applicability of these results in different groups somewhat biased, so we need to interpret these data with caution.

The findings underscore the superiority of combination therapy over single-agent treatments in enhancing both ORR and DCR in the treatment of solid tumors. However, the study’s assessment of 12-month OS (45.2%) and PFS rate (18.9%) fell short of expectations when compared to a phase 2 clinical trial of camrelizumab monotherapy for advanced hepatocellular carcinoma (OS: 55.9%) (61), The results of the analyses of OS and PFS showed that although camrelizumab in combination with apatinib was effective in delaying disease progression and improving survival in the short term, patients’ survival and disease progression progressively worsened as the duration of treatment increased. This was particularly evident in the 12- and 24-month PFS and OS results. The higher PFS in the short term (6 months) suggests that most patients were able to achieve better efficacy control in the early stages of treatment, however, the rate of disease progression increased significantly over time. In addition, although PFS showed better control in the short term, the significant decline in long-term PFS suggests that the treatment regimen may have issues with drug resistance or that the efficacy of the treatment is diminishing over time. The long-term PFS and OS results highlight the need to further optimize treatment strategies and improve long-term patient survival. Overall, subgroup analyses of both OS and PFS showed high heterogeneity (I² values of more than 75% in both cases), a phenomenon that may stem from several factors, including different patient populations (different tumor types, different clinical manifestations), different treatment modalities, and differences in study design. This suggests the need for caution in interpreting the results, and further studies may be needed to refine the effects of different factors on efficacy. In addition, given the high degree of heterogeneity, future studies should attempt to identify the key factors affecting patient survival and disease progression, thus providing guidance for individualized treatment. Although the 24-month OS and PFS results were low, this may be related to the severity of the patients’ baseline disease and the impact of treatment continuity, indicating that further optimization is needed in patient selection and treatment strategies.

In this study, diarrhea, anemia, and hypertension emerged as prevalent adverse effects associated with apatinib, consistent with its known safety profile. These adverse effects likely stem from apatinib inhibition of the Vascular Endothelial Growth Factor (VEGF) and VEGFRs pathways, which are prominently expressed in intestinal endothelial cells (64). A study involving 207 patients with Hepatocellular Carcinoma (HCC) treated with apatinib reported an incidence of all-grade diarrhea of 22.7%, with only 1.0% experiencing grade 3 or higher diarrhea. Another study focusing on PD-1/PD-L1-related adverse effects found an all-grade diarrhea incidence of 9.47%, with grade 3 or higher diarrhea occurring in 0.59% of cases (65). Comparing the incidence of diarrhea associated with the combination therapy to that of single agent apatinib, it was observed that the combination therapy led to an elevated incidence of diarrhea. These adverse events may significantly impact patients’ quality of life and pose challenges for clinical management. Therefore, future clinical applications should place particular emphasis on managing these side effects, especially during long-term treatment. We did not document whether these adverse events led to dose adjustments or treatment discontinuation. Future studies should explore the relationship between adverse events and treatment dose adjustments or discontinuation to comprehensively assess the risks and benefits of treatment. Although this study demonstrated efficacy, the high incidence of side effects may impact patients’ quality of life, suggesting the need for a more precise balance between risks and benefits. Therefore, future studies should further focus on management strategies for these adverse effects and explore their relationship with efficacy to inform clinical decision-making. To optimize treatment regimens, it is recommended to conduct detailed monitoring and intervention for adverse effects such as hypertension, proteinuria, and anemia, assess the impact of different intervention measures on improvements in patients’ quality of life, and systematically analyze the influence of adverse events on treatment outcomes and adjustments to treatment strategies.

Overall, although the single-arm design studies in this research provided preliminary efficacy data, the absence of a control group precludes the complete exclusion of confounding factors, such as patients’ underlying diseases and differences in treatment responses. Therefore, the interpretation of these study results should consider these potential confounding factors. We recommend that future studies adopt a randomized controlled design or apply statistical adjustment methods (such as multivariate regression analysis) in single-arm studies to better control confounding factors and provide more reliable efficacy evaluations.

Although the introduction section provides a detailed explanation of the synergistic mechanism of the combination therapy of camrelizumab and apatinib, the clinical results of this study do not fully support this theory. While the survival curves and DCR we observed demonstrate some clinical efficacy, the survival outcomes, particularly the sharp decline in OS at 24 months, fail to fully reflect the synergistic effects anticipated in the theoretical model. This may be attributed to patient heterogeneity, differences in treatment adherence, and other uncontrolled variables. Therefore, while the theoretical mechanism provides a reasonable basis for treatment, these clinical outcomes indicate that there remains a gap between actual efficacy and theoretical effects. Future studies should further validate these mechanisms and assess whether other factors may have influenced clinical efficacy.

The current study still has the following limitations: first, most of the studies were single-arm, single-center and lacked a control group, which these results may be influenced by baseline characteristics, comorbidities, or other treatment factors. Therefore, these results should be interpreted with caution, and future studies should consider using control groups or adjusting for these confounding factors using statistical methods; second, the inclusion of the sample size was small, the determinability of the efficacy was not high, the patients with intermediate and advanced stages were more numerous, the follow-up time was shorter, and it was the result of a preliminary trial, therefore, it might affect the representativeness of the data and cause a certain bias.

## 5 Conclusion

According to the results of this meta-analysis, although the camrelizumab plus apatinib treatment regimen demonstrated certain efficacy in the short term, due to the significant limitations of this study, more high-quality, multicenter, large sample randomized controlled studies are needed in the future to corroborate our conclusions.

## Data Availability

The original contributions presented in the study are included in the article/**Supplementary Material**. Further inquiries can be directed to the corresponding authors.
