# Correction: Efficacy and safety of camrelizumab plus apatinib for solid tumors: a meta-analysis

**DOI:** 10.3389/fimmu.2026.1987050

**Published:** 2026-09-28

**Authors:** Ping Yu, Xiaoyu Song, Yue Li, Xuanlong Chen, Yuan Zhou, Fei Jiang

**Affiliations:** 1 Department of Pharmacy, Hubei Provincial Clinical Research Center for Umbilical Cord Blood Hematopoietic Stem Cells, Taihe Hospital, Hubei University of Medicine, Shiyan, Hubei, China; 2 Departmentof Ultrasound Medicine, Taihe Hospital, Hubei University of Medicine, Shiyan, Hubei, China; 3 Department of Respiratory, Taihe Hospital of Shiyan, Hubei University of Medicine, Shiyan, Hubei, China

**Keywords:** camrelizumab, apatinib, solid tumors, meta-analysis, efficacy

In the published article, an incorrect **Funding** statement was provided. The statement read: “The author(s) declare that no financial support was received for the research and/or publication of this article.”

The **Funding** statement has been corrected to read: “The author(s) declare that financial support was received for the research and/or publication of this article. This research was funded by a special grant for stem cell research from Taihe Hospital in Shiyan (GXB23015) and Natural Science Foundation of Hubei Province, China (2024AFB848).”

The original version of this article has been updated.

