# Supplementary material for: Efficacy and safety of camrelizumab plus apatinib for solid tumors: a meta-analysis
Source: Front Immunol. 2025 Sep 2;16:1653429. doi: 10.3389/fimmu.2025.1653429 (PMC12436330; doi:10.3389/fimmu.2025.1653429)
Supplement: Supplementary file 1 [file DataSheet1.docx]

Table S1 Search history

| Database | search history |
| --- | --- |
| PubMed | (((((camrelizumab[Title/Abstract]) OR (carrelizumab[Title/Abstract])) OR (SHR-1210[Title/Abstract])) OR (SHR 1210[Title/Abstract])) AND (("apatinib" [Supplementary Concept]) OR ((((((apatinib[Title/Abstract]) OR (rivoceranib mesylate[Title/Abstract])) OR (YN968D1[Title/Abstract])) OR (YN-968D1[Title/Abstract])) OR (rivoceranib[Title/Abstract])) OR (apatinib mesylate[Title/Abstract])))) AND (("Neoplasms"[Mesh]) OR ((((((((((((((((((Neoplasms[Title/Abstract]) OR (Tumor[Title/Abstract])) OR (Neoplasm[Title/Abstract])) OR (Tumors[Title/Abstract])) OR (Neoplasia[Title/Abstract])) OR (Neoplasias[Title/Abstract])) OR (Cancer[Title/Abstract])) OR (Cancers[Title/Abstract])) OR (Malignant Neoplasm[Title/Abstract])) OR (Malignancy[Title/Abstract])) OR (Malignancies[Title/Abstract])) OR (Malignant Neoplasms[Title/Abstract])) OR (Neoplasm, Malignant[Title/Abstract])) OR (Neoplasms, Malignant[Title/Abstract])) OR (Benign Neoplasms[Title/Abstract])) OR (Benign Neoplasm[Title/Abstract])) OR (Neoplasms, Benign[Title/Abstract])) OR (Neoplasm, Benign[Title/Abstract]))) |
| Embase | \| #35 \| #6 AND #14 AND #34 \| \| --- \| --- \| \| #34 \| #15 OR #16 OR #17 OR #18 OR #19 OR #20 OR #21 OR #22 OR #23 OR #24 OR #25 OR #26 OR #27 OR #28 OR #29 OR #30 OR #31 OR #32 OR #33 \| \| #33 \| 'neoplasm, benign':ab,ti \| \| #32 \| 'neoplasms, benign':ab,ti \| \| #31 \| 'benign neoplasm':ab,ti \| \| #30 \| 'benign neoplasms':ab,ti \| \| #29 \| 'neoplasms, malignant':ab,ti \| \| #28 \| 'neoplasm, malignant':ab,ti \| \| #27 \| 'malignant neoplasms':ab,ti \| \| #26 \| 'mmalignancies':ab,ti \| \| #25 \| 'malignancy':ab,ti \| \| #24 \| 'malignant neoplasm':ab,ti \| \| #23 \| 'cancers':ab,ti \| \| #22 \| 'cancer':ab,ti \| \| #21 \| 'neoplasias':ab,ti \| \| #20 \| 'neoplasia':ab,ti \| \| #19 \| 'tumors':ab,ti \| \| #18 \| 'neoplasm':ab,ti \| \| #17 \| 'tumor':ab,ti \| \| #16 \| 'neoplasms':ab,ti \| \| #15 \| 'neoplasm'/exp \| \| #14 \| #7 OR #8 OR #9 OR #10 OR #11 OR #12 OR #13 \| \| #13 \| 'apatinib mesylate':ab,ti \| \| #12 \| 'yrivoceranib':ab,ti \| \| #11 \| 'yn-968d1':ab,ti \| \| #10 \| 'yn968d1':ab,ti \| \| #9 \| 'rivoceranib':ab,ti \| \| #8 \| 'apatinib':ab,ti \| \| #7 \| 'rivoceranib'/exp \| \| #6 \| #1 OR #2 OR #3 OR #4 OR #5 \| \| #5 \| 'shr 1210':ab,ti \| \| #4 \| 'shr-1210':ab,ti \| \| #3 \| 'carrelizumab':ab,ti \| \| #2 \| 'camrelizumab':ab,ti \| \| #1 \| 'camrelizumab'/exp \| |
| Cochrane | ID Search Hits  #1 MeSH descriptor: [camrelizumab] explode all trees  #2 (camrelizumab):ti,ab,kw OR (carrelizumab):ti,ab,kw OR (SHR-1210):ti,ab,kw OR (SHR 1210):ti,ab,kw  #3 #1or#2  #4 MeSH descriptor: [apatinib] explode all trees  #5 (apatinib):ti,ab,kw OR (rivoceranib mesylate):ti,ab,kw OR (YN968D1):ti,ab,kw OR (YN-968D1):ti,ab,kw OR (rivoceranib):ti,ab,kw  #6 (apatinib mesylate):ti,ab,kw  #7 #4or#5or#6  #8 MeSH descriptor: [Neoplasms] explode all trees  #9 (Neoplasms):ti,ab,kw OR (Tumor):ti,ab,kw OR (Neoplasm):ti,ab,kw OR (Tumors):ti,ab,kw OR (Neoplasia):ti,ab,kw  #10 (Neoplasias):ti,ab,kw OR (Cancer):ti,ab,kw OR (Cancers):ti,ab,kw OR (Malignant Neoplasm):ti,ab,kw OR (Malignancy):ti,ab,kw  #11 (Malignancies):ti,ab,kw OR (Malignant Neoplasms):ti,ab,kw OR (Neoplasm, Malignant):ti,ab,kw OR (Neoplasms, Malignant):ti,ab,kw OR (Benign Neoplasms):ti,ab,kw  #12 (Benign Neoplasm):ti,ab,kw OR (Neoplasms, Benign):ti,ab,kw OR (Neoplasm, Benign):ti,ab,kw  #13 #8or#9or#10or#11or#12  #14 #3and#7and#13 |
| Web of science | \| 1 \| TS=(camrelizumab) OR TS=(carrelizumab) OR TS=(SHR-1210) OR TS=(SHR 1210) \| \| --- \| --- \| \| 2 \| TS=(apatinib) OR TS=(rivoceranib mesylate) OR TS=(YN968D1) OR TS=(YN-968D1) OR TS=(rivoceranib) OR TS=(apatinib mesylate) \| \| 3 \| TS=(Neoplasms) OR TS=(Tumor) OR TS=(Neoplasm) OR TS=(Tumors) OR TS=(Neoplasia) OR TS=(Neoplasias) OR TS=(Cancer) OR TS=(Cancers) OR TS=(Malignant Neoplasm) OR TS=(Malignancy) OR TS=(Malignancies) OR TS=(Malignant Neoplasms) OR TS=(Neoplasm, Malignant) OR TS=(Neoplasms, Malignant) OR TS=(Benign Neoplasms) OR TS=(Benign Neoplasm) OR TS=(Neoplasms, Benign) OR TS=(Neoplasm, Benign) \| \| 4 \| #1 AND #2 AND #3 \| |

Table S2: Results of grade ratings

| Outcome | grade |
| --- | --- |
| Adverse events | Moderate |
| Objective Response Rate | Moderate |
| Disease Control Rate | Moderate |
| Overall Survival | Moderate |
| Progression-Free Survival | Moderate |

Table S3: Newcastle-Ottawa Scale (NOS) for studies

| Study | Selection | Comparability | Exposure | Total score |
| --- | --- | --- | --- | --- |
| Chen2024 | ******* | ****** | ****** | 7 |
| Cheng2021 | ****** | ****** | ****** | 6 |
| Ding2023 | ******* | ***** | ****** | 6 |
| Gao2022 | ******* | ****** | ******* | 8 |
| Ju2022 | ******* | ****** | ****** | 7 |
| Liang2024 | ****** | ****** | ******* | 7 |
| Ma2022 | ******* | ****** | ******* | 8 |
| Mei2021 | ******* | ****** | ****** | 7 |
| Meng2022 | ****** | ****** | ****** | 6 |
| Mo2023 | ******* | ***** | ****** | 6 |
| Qu2024 | ******* | ****** | ******* | 8 |
| Ren2024 | ******* | ****** | ****** | 7 |
| Wang2021 | ****** | ****** | ******* | 7 |
| Wang2023 | ******* | ****** | ******* | 8 |
| Xia2022 | ******* | ****** | ****** | 7 |
| Xu2021 | ****** | ****** | ****** | 6 |
| Yao2023 | ******* | ***** | ****** | 6 |
| Yuan2020 | ******* | ****** | ******* | 8 |
| Yuan2023 | ******* | ****** | ****** | 7 |
| Zeng2021 | ****** | ****** | ******* | 7 |
| Zhang2020 | ******* | ****** | ******* | 8 |
| Zhou2021 | ******* | ****** | ******* | 8 |
| Fan2021 | ****** | ****** | ****** | 6 |
| Lan2020 | ******* | ***** | ******* | 7 |
| Tian2024 | ******* | ****** | ******* | 8 |
| Xia2024 | ******* | ****** | ******* | 8 |
| Yu2024 | ****** | ****** | ****** | 6 |
| Zhao2024 | ******* | ***** | ******* | 7 |

Table S4 Results of meta regression analysis

| Outcomes | Group | P |
| --- | --- | --- |
| OS | Type of tumor | 0.001 |
|  | Front line | 0.45 |
| PFS | Type of tumor | 0.02 |
|  | Front line | 0.83 |
| ORR | Type of tumor | 0.04 |
|  | Front line | 0.17 |
| DCR | Type of tumor | 0.01 |
|  | Front line | 0.09 |


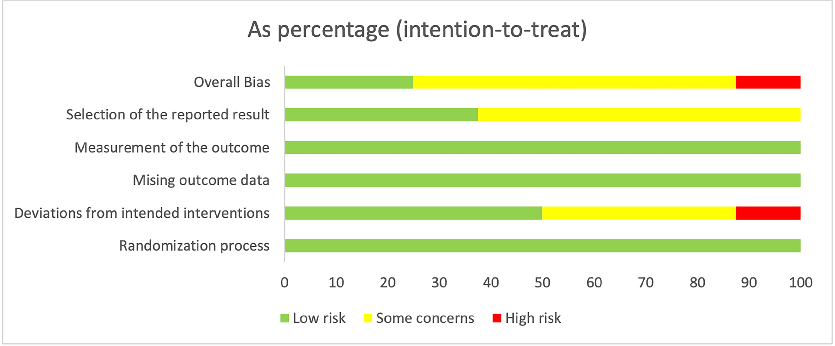


Figure S1 Risk of bias results


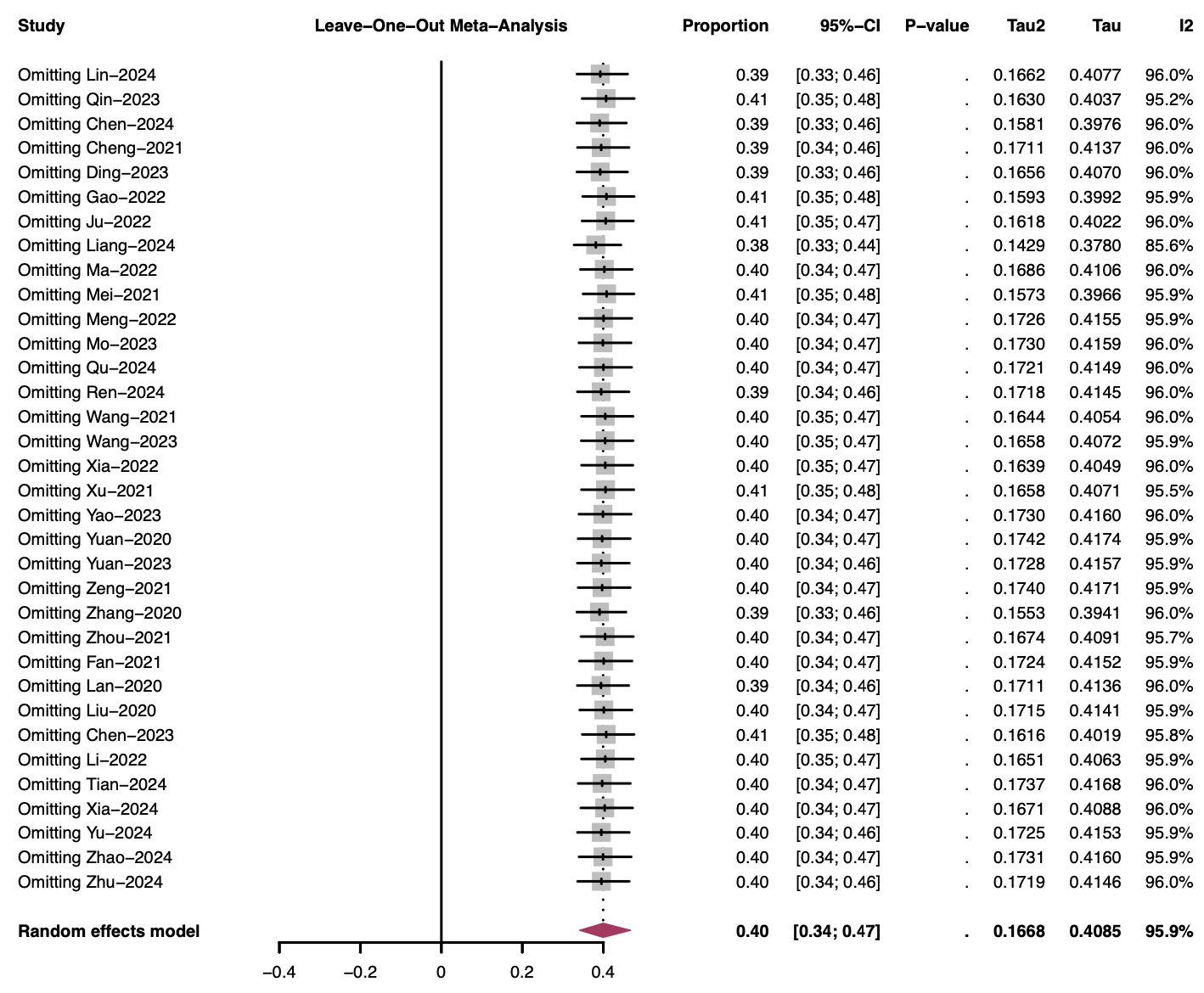


Figure S2 Objective response rate sensitivity analysis


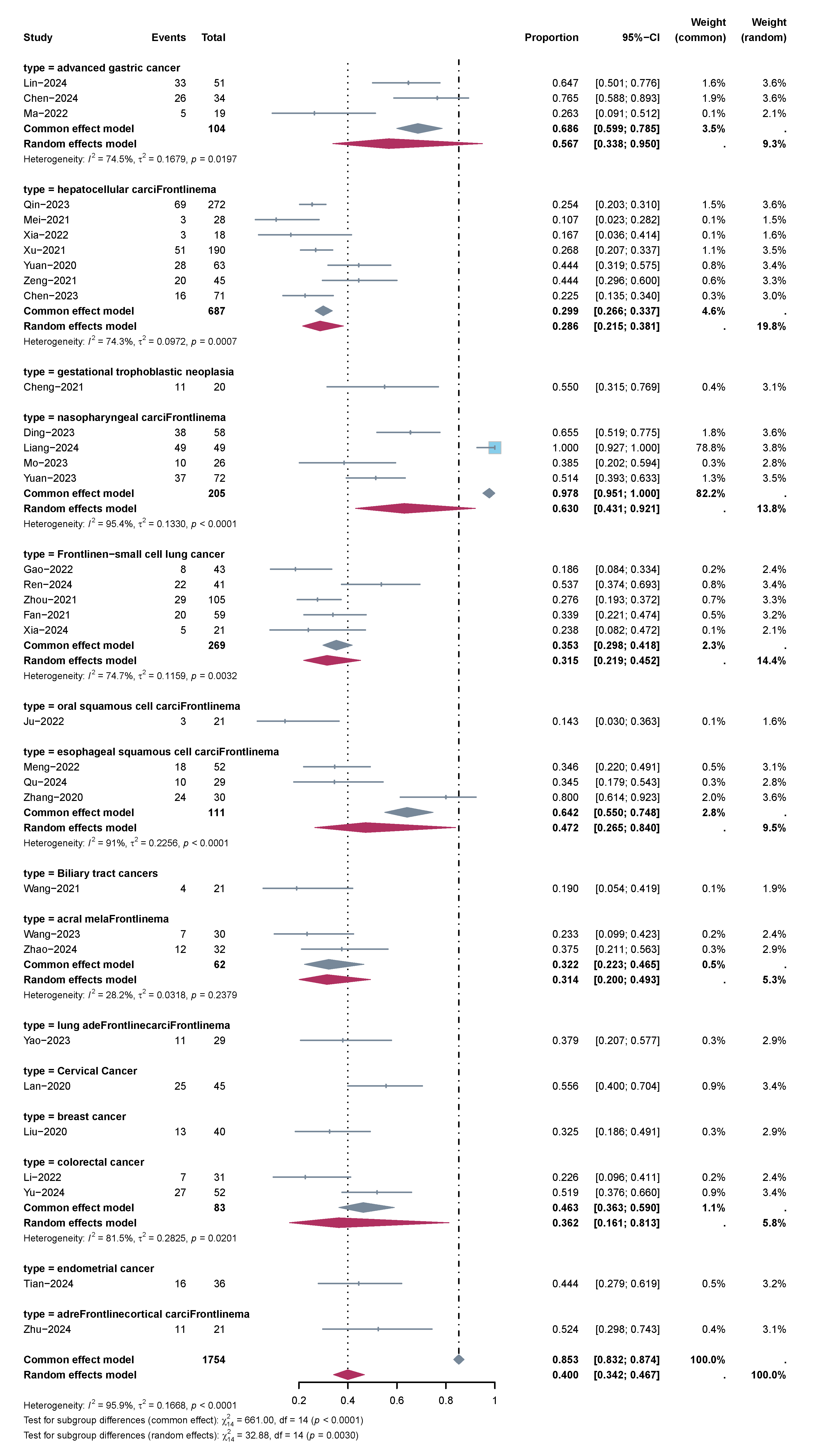


Figure S3 Objective response rate type of tumor subgroup analysis


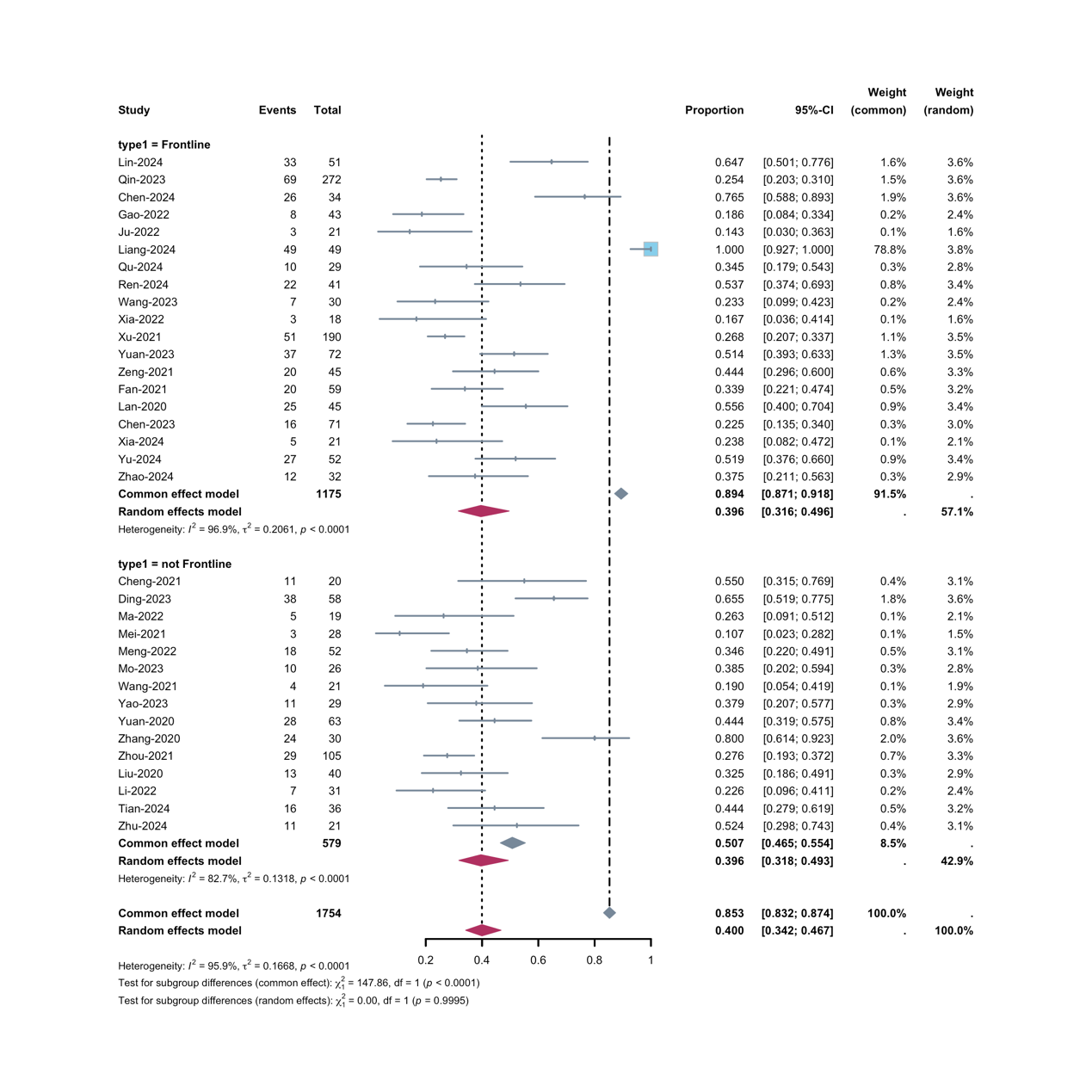
Figure S4 Objective response rate different lines of treatment subgroup analysis

Figure S5 Disease control rate sensitivity analysis


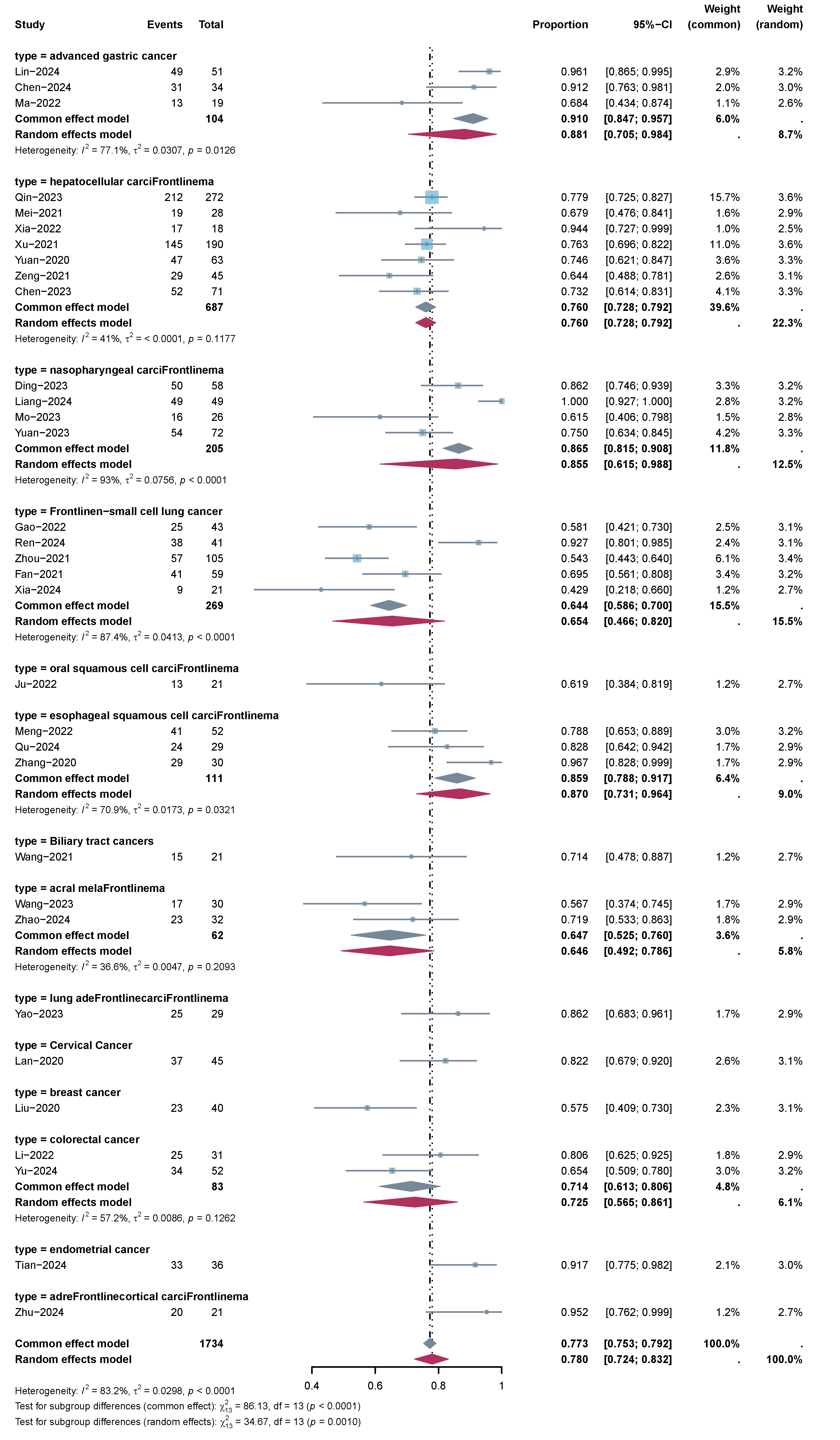


Figure S6 Disease control rate type of tumor subgroup analysis

Figure S7 Disease control rate First-line treatment subgroup analysis

Figure S8 Disease control rate publication bias

Figure S9 Objective response rate publication bias

Figure S10 Overall survival publication bias

Figure S11 Progression free survival publication bias
